# Calcium Alkali Thiazide Syndrome: What We Need to Know

**DOI:** 10.7759/cureus.10856

**Published:** 2020-10-08

**Authors:** Mehboob A Rehan, Asma Rashid, Kenneth Krell, Cristina Gabutti, Reema Singh

**Affiliations:** 1 Department of Medicine, Eastern Idaho Regional Medical Center, Idaho Falls, USA; 2 Department of Critical Care Medicine, Eastern Idaho Regional Medical Center, Idaho Falls, USA

**Keywords:** hypercalcemia, thiazide diuretics in elderly, acute kidney injury and alkalosis, calcium alkali thiazide syndrome, parallel testing vs sequential testing, sippy diet, milk alkali syndrome, calcium alkali syndrome

## Abstract

Depending on each institution's laboratory test, mean serum calcium levels range between 8.8 and 10.8 mg/dL and hypercalcemia is defined as two standard deviations above the mean. According to recent epidemiological studies, 90% of cases of hypercalcemia are due to hyperparathyroidism or malignancy. Milk Alkali syndrome (MAS) also known as Calcium Alkali syndrome (CAS) is the third biggest cause of hypercalcemia, but its incidence seems to be higher than previously thought. Here we present a case of Calcium Alkali Thiazide syndrome (CATS) in a 57-year-old female who was on calcium and vitamin D supplements (after parathyroidectomy) while also taking thiazide diuretic for hypertension. She was brought to the ED with nausea, vomiting, confusion, difficulty walking along with numbness in extremities. She had parathyroidectomy three weeks ago. During history taking, patient reported intake of calcium carbonate 1 g three times daily, calcitriol 0.5 mcg twice daily, cholecalciferol (vitamin D3) 10,000 units once daily, chlorthalidone 25 mg once daily and irbesartan 300 mg once daily. At admission, her calcium level was 23 mg/dL, ionized calcium 12.03 mg/dL, pH was 7.59 and HCO3 was 33. She was in renal failure with creatinine of 1.9 mg/dL (baseline 0.8 mg/dL). Her parathyroid hormone (PTH) level was 0. A diagnosis of CATS was made. She was treated with intravenous fluids and furosemide and discharged home on hospital day 5 after her calcium and creatinine levels normalized. A triad of hypercalcemia, acute kidney injury and metabolic alkalosis comprises MAS. Traditional MAS was caused by "Sippy diet" (containing milk and alkali) used for the treatment of peptic ulcer disease. Over the decades, the same triad of symptoms occurred in patients using excess calcium and vitamin D, hence changing the name to CAS. A subset of patients at risk for CAS also use thiazide diuretics for hypertension, making them more vulnerable to hypercalcemia and acute kidney injury. In such subset of patients, it is preferable to use the term CATS rather than MAS or CAS.

## Introduction

Calcium level above 10.5 mg/dL is considered abnormal but depending on each institution's laboratory, mean serum calcium levels can range from 8.8 to 10.8 mg/dL. Hypercalcemia is defined as calcium levels two standard deviations above the mean. Hypercalcemia prevalence in the ED ranges between 1% and 2%. Hyperparathyroidism and malignancy account for 90% of these cases [1]. Multiple myeloma is the most common malignancy causing hypercalcemia in 7.5%-10.2% of cases [1,2]. It is essential to know the differential diagnosis of hypercalcemia, as sequential testing will often lead to an efficient diagnosis without a futile search for underlying malignancy. Many cases of iatrogenic hypercalcemia are missed due to poor history taking from the patient or inaccurate utilization of information in clinical practice. We present here an interesting case of Calcium Alkali syndrome (CAS) in a patient who was taking an excessive amount of calcium and vitamin D (vitamin D) while concurrently being treated with thiazide diuretic for hypertension. Original term of Milk Alkali syndrome (MAS) was used for a triad of symptoms, hypercalcemia, acute kidney injury (AKI) and metabolic alkalosis which resulted from "Sippy diet" used for peptic ulcer disease (PUD) in the early 1900s. Since 1970, H2 blockers and proton pump inhibitors replaced "Sippy diet" as treatment of PUD, but the same triad of symptoms occurred in patients using excessive calcium and vitamin D (especially for prevention and treatment of osteoporosis), so MAS was replaced by a better term CAS. A subset of CAS patients are also on thiazide diuretics for hypertension making them more vulnerable to hypercalcemia, hence we devised a new term Calcium Alkali Thiazide syndrome (CATS) for such subset of patients. 

## Case presentation

A 57-year-old lady was brought to the ED by family with complaints of severe generalized body weakness, confusion, difficulty walking, achiness in legs, numbness in extremities, dizziness, nausea, vomiting, abdominal pain, constipation and occasional palpitations. Patient had total parathyroidectomy and partial thyroidectomy three weeks ago for primary hyperparathyroidism caused by parathyroid adenomas. Serum calcium level at the referring facility was 21 mg/dL. EKG showed prolonged QTc of 476 ms and nonspecific ST-T wave changes, she denied chest pain and troponin levels were <0.015. At the time of admission in our facility, her vital signs were temperature 97.9 F, heart rate 87 bpm, respiratory rate 16/min and blood pressure 163/67 mmHg. She was alert and oriented, had normal reflexes through symmetrically decreased muscle strength in all four extremities with mild generalized abdominal tenderness. Rest of the physical exam was unremarkable. Patient’s initial laboratory values showed white blood cell (WBC) count of 15.5 k/mm^3,^ hemoglobin (Hb) of 15.6 gm/dL, platelet count of 370 k/mm^3^, creatinine of 1.9 mg/dL (baseline was 0.8), BUN of 45 mg/dL, eGFR of 27 mL/min (baseline eGFR >60 mL/min), bicarbonate of 33 mEq/L, chloride of 94 mEq/L, potassium of 2.5 mEq/L, sodium of 135 mEq/L, total calcium of 23 mg/dL with ionized calcium of 12.03 mg/dL, phosphate of 1.3 mg/dL and PTH of 0. Her 25 hydroxyvitamin D level was 61 ng/mL (normal 30-100 ng/mL) and 1-25 hydroxyvitamin D3 level was 31 pg/ml (normal 18-72 pg/mL) (Table 1).

**Table 1:** Patients relevant labs from admission to discharge

Labs	Day 1	Day 2	Day 3	Day 4	Day 5	Day 6
Calcium (mg/dL)	23	17.9	14.7	14.5	11.7	10.3
Ionized Calcium (mg/dL)	12.03	9.26	8.76		6.48	5.77
Creatinine (mg/dL)	1.9	1.9	1.7	1.9	1.5	1.4
eGFR (mL/min)	27	27	31	27	36	39
HCO3 (MEQ/L)	33	31	30	28	25	26
pH (7.32-7.42)	7.59	7.47	7.46		7.47	7.42
Phosphate (mg/dL)	1.3	5.7			2.7	

When asked about medication history, patient reported 3 g calcium carbonate intake daily, calcitriol 0.5 mcg twice daily, cholecalciferol (vitamin D3) 10,000 units once daily, chlorthalidone 25 mg once daily and irbesartan 300 mg once daily. Her diet included a glass of milk after each meal. As explained earlier, a diagnosis of CATS was made, and treatment was started. It was obvious from history that hypercalcemia was due to excessive calcium intake so calcium, vitamin D supplements and chlorthalidone were stopped. Irbesartan contributing to AKI was also stopped. Aggressive hydration was instituted with intravenous (IV) normal saline 150-200 mL/hour with 4L given in the first 24 hours. Furosemide 40 mg twice daily was also started. Patient didn’t require calcitonin. Due to the anticipated serious risk of late hypocalcemia owing to parathyroidectomy, bisphosphonates were not considered. Patient improved clinically and her symptoms resolved, she was discharged on hospital day 5, with total calcium level reduced to 10.3 mg/dL and ionized calcium to 5.77 mg/dL. Her creatinine levels were close to baseline at 1.4 mg/dL on the day of discharge. 

## Discussion

AKI is almost always associated with metabolic acidosis. Some of the most common causes of increased anion gap metabolic acidosis are AKI, sepsis and diabetic ketoacidosis [3]. But, what if AKI is associated with alkalosis and not acidosis? Interesting isn’t it? What if elevated bicarbonate and creatinine level are also associated with hypercalcemia? The triad of metabolic alkalosis, hypercalcemia and acute kidney injury are the hallmark of MAS/CAS, which is regarded as the third most common cause of hypercalcemia after hyperparathyroidism and malignancy [4]. Incidence of MAS/CAS has been on the rise since 1970s due to the widespread use of calcium carbonate and vitamin D. CAS is more common in females who use calcium and vitamin D for prevention and treatment of osteoporosis and also in patients with chronic kidney disease who use calcium carbonate for prevention of secondary hyperphosphatemia [4,5]. In our literature review, we couldn’t find any case of severe hypercalcemia occurring after total or partial parathyroidectomy. Our case highlights the importance of diagnosing CATS in patients who are using calcium and vitamin/ D supplements, while also concurrently on thiazide diuretics for hypertension making them vulnerable to hypercalcemia and subsequent renal failure. We believe that AKI in our patient was the result of hypercalcemia which was potentiated by the use of aldosterone receptor blocker (ARB) irbesartan, and was stopped immediately.

It is important to know the history of MAS. It all started in 1910, when PUD information was scarce in medical literature until British surgeon Sir Berkeley Moynihan pointed out the high incidence of duodenal ulcer. In 1912, a brilliant diagnostician, Bertram Welton Sippy, without the aid of invasive radiological procedures and expensive lab tests concluded that “the pain and the discomfort of uncomplicated ulcer are due to the irritative action of hydrochloric acid on the nerves exposed in the ulcer” [6]. This was the basis of popular “Sippy diet” for the treatment of PUD which included hourly administration of milk and cream together with Sippy powders (10 grains of heavily calcinated magnesia and sodium bicarbonate alternating with 10 grains of bismuth subcarbonate and 20-30 grains of sodium bicarbonate, each grain is equal to 65 mg) [7,8]. Until the discovery of H2 blockers and proton pump inhibitors, Sippy diet and other forms of antacid therapy remained the cornerstone therapy for PUD [7]. Hardly did Dr. Bertram know that after 100 years, his Sippy diet would be mentioned, not in the treatment of PUD but as a cause of MAS. Hard and River first studied the toxic effects of calcium and alkali and later in 1936 Cope described the classic triad of hypercalcemia, metabolic alkalosis and renal failure of MAS [7,9]. Many authors in the past decade have recommended changing the name to CAS as it reflects the changing epidemiology and the cause, which is no longer the Sippy diet [9]. The case was entirely different in our patient as she was taking huge amounts of calcium tablets and vitamin D along with consumption of milk while on thiazide diuretic to prevent hypocalcemia after parathyroid removal. This case also reflects the importance of good history taking as any calcium level above 14 mg/dl usually alerts the physician to pursue a malignancy workup, initiating an extensive evaluation.

Three different types of MAS/CAS have been described: acute, subacute and chronic. The acute form occurs days to weeks after starting calcium supplements, the subacute form, also known as Cope’s syndrome, tends to occur when calcium and alkali are used intermittently for years and the chronic form (Burnett’s syndrome) occurs when a huge amount of calcium and alkali are used over years. Patients can have soft tissue calcium deposition along with kidney stones and band keratopathy [7,9]. It is important to note that classically 4 g of calcium intake daily is required to develop acute MAS, but there are cases that reported MAS in patients taking only 1-1.5 g of daily calcium [8,10]. Maximum calcium levels reached in serum of patients with MAS/CAS are controversial but some authors report that they don't exceed 16-17 mg/dL [5]. In our patient calcium level exceeded 23 mg/dL, maybe due to co-administration of the thiazide diuretic. Extremely high abnormal calcium levels have been reported in the past from parathyroid adenomas. Keeling et al. [11], Basok et al. [12] and Marienhagen et al. [13] reported calcium levels of 30.46 mg/dL, 23.9 mg/dL and 27.7 mg/dL, respectively, in their patients who all had primary hyperparathyroidism from parathyroid adenoma. There is one very important difference between traditional MAS and new CAS which needs clarification. Traditional MAS was caused by Sippy diet which leads to hyperphosphatemia due to phosphate-rich milk and cream in the Sippy diet. As no extra milk and cream are taken in the modern CAS, hypophosphatemia or normal phosphorus levels are found, caused by phosphate binding capacity of calcium carbonate which decreases absorption of phosphate from the gut [14]. This was evident in our patient as well, whose initial phosphate level was 1.3 mg/dL. For patients with CAS, their calcium level will decrease during hospitalization when all calcium-raising medications are stopped. This would be in contrast to hormonal and lytic causes of hypercalcemia, which will continue to rise or stay the same, despite cessation of calcium-raising therapy.

Acute form of MAS/CAS develops after at least one week of extra calcium intake with patients commonly presenting with nausea, vomiting and confusion. Several different mechanisms cause kidney injury in hypercalcemia. These mechanisms include renal afferent arteriolar vasoconstriction resulting in decreased glomerular filtration rate (GFR) which further increases serum calcium levels. Hypercalcemia also leads to a reversible decrease in the ability of the kidney to concentrate urine, especially at calcium levels exceeding 11 mg/dL, resulting in nephrogenic diabetes insipidus which in turn causes a cycle of renal vasoconstriction, distal renal tubular acidosis and polyuria. If calcium remains chronically elevated, kidney stones, renal tubular dysfunction and chronic kidney failure ensues [15-17]. In our patient, irbesartan contributed to AKI in addition to hypercalcemia.

This case also gives us the opportunity to discuss different causes of hypercalcemia. In one study, 44% of patients with hypercalcemia in the ED had underlying malignancy, secondary hyperparathyroidism was found in 12% and primary hyperparathyroidism in 8% [18]. The laboratory values in our patient demonstrated a high calcium, low PTH, low phosphate, normal vitamin D [25-(OH) D] and vitamin D3 [1,25-(OH)2D] with unremarkable PTHrP. This leaves us with a differential diagnosis of MAS/CAS, hypercalcemia caused by thiazide, familial hypocalciuric hypercalcemia or increased bone turnover. On the contrary, vitamin D excess or toxicity would have led to increased phosphate and increased vitamin D. In primary and tertiary hyperparathyroidism, we would have seen an elevated PTH. Elevated calcium levels of 23 mg/dL would have been unlikely with familial hypocalciuric hypercalcemia. Several mechanisms can cause hypercalcemia in malignancies, in 80% of cases parathyroid hormone-related protein (PTHrP) acts on osteoblasts causing increased expression of receptor activator of nuclear factor kappa-B ligand (RANKL) ^ ^ultimately leading to activation of osteoclasts, bone resorption and increased reabsorption of calcium from kidneys causing hypercalcemia [19]. In 20% of cases, osteolytic metastasis (breast, multiple myeloma) causes hypercalcemia via bone resorption (Table 2).

**Table 2:** Conditions with high calcium and low PTH PTH, parathyroid hormone

Diseases Labs	Malignancy	Calcium Alkali Syndrome And Thiazides (CATS)	Familial Hypocalciuric Hypercalcemia	Hyperparathyroidism (1^o^ and 3^o^)	Vitamin D excess	Chronic Renal Failure (2^o ^Hyperparathyroidism)
Calcium	High	High	High	High	High	Low
PTH	Low (PTHrP high)	Low	Increased or Normal	High	Low	High
PO_4_	Low	Low	Low	Low	High	High
25-(OH)D	Low to Normal	Normal	Normal	Low to Normal	High	Variable
1,25-(OH)_2_D	High	Normal	Normal	High	Variable	Decreased

Based on the literature review, our patient falls under the category of acute CAS but we prefer to call it CATS due to concurrent intake of chlorthalidone, a thiazide diuretic. This is probably the only case in the literature of CAS/CATS where calcium levels of more than 23 mg/dL were recorded. Our case also underlines the importance of taking medication history including over-the-counter (OTC) use of calcium/vitamin D or other supplements before starting thiazide diuretic in patients with hypertension.

## Conclusions

Our case highlights many important aspects of practicing medicine. First, the importance of good history taking including information about prescription medications and the use of OTC drugs earlier in the course of evaluation. Second, the significance of sequential testing, which can lower enormous healthcare-related costs. Finally, caution and consideration about drug interaction while prescribing medications such as thiazide diuretics with supplements such as calcium/vitamin D along with medications that can cause AKI (in our patient’s case irbesartan). Our observation is that a high proportion of the general population uses nutritional supplements which, when combined with prescription medications, can cause electrolyte imbalances and renal insult. This poses a dilemma about hypercalcemia cases due to CAS and CATS that are missed, resulting in unnecessary costly testing and misrepresentation about true incidence and prevalence of this syndrome. That also leads us to believe that the true incidence of CAS and CATS is not known as many patients are under or misdiagnosed. Large scale epidemiological studies are needed in this regard to know the true incidence of CAS and CATS.
